# An Evaluation of the Second Trimester Thyroid Function Test in Gestational Diabetes Mellitus: A Case-Control Study

**DOI:** 10.7759/cureus.41858

**Published:** 2023-07-13

**Authors:** Himsweta Srivastava, Priyanka Mathe, Nibedita Mondal, Sushil Srivastava, SV Madhu, Rachna Agarwal

**Affiliations:** 1 Department of Obstetrics and Gynaecology, University College of Medical Sciences, New Delhi, IND; 2 Department of Obstetrics and Gynaecology, Dr. Hedgewar Arogya Sansthan, New Delhi, IND; 3 Department of Paediatrics, University College of Medical Sciences, New Delhi, IND; 4 Department of Endocrinology, University College of Medical Sciences, New Delhi, IND

**Keywords:** thyroid hormone, insulin resistance, hypothyroidism, gdm, gestational diabetes mellitus

## Abstract

Introduction: Gestational diabetes mellitus (GDM) is defined as glucose intolerance in a female with its onset or first recognition during pregnancy. Females with GDM are at higher risk of developing antenatal complications like preeclampsia during pregnancy and increased risk of type 2 diabetes as well as cardiovascular disorders later in their life. Maternal thyroid changes in the first and second trimesters of pregnancy have been widely related to the risk of GDM. Hypothyroidism during pregnancy is associated with early and late complications like abortions, anaemia, gestational hypertension, placental abruption and postpartum haemorrhage, impaired infant neurodevelopment, and low birth weight.

Objectives: This study aims to compare the thyroid function test (TFT) (serum fT3, fT4, TSH) and thyroid peroxidase antibody (anti TPO) between GDM and non GDM pregnant women in the second trimester and to correlate the adverse pregnancy outcomes with TFT in GDM and non GDM women.

Methods: A nested case-control study was done in the Department of Obstetrics and Gynaecology, Department of Endocrinology, Department of Paediatrics, University College of Medical Sciences, and Guru Teg Bahadur (GTB) Hospital, Delhi. About 350 pregnant women from 13 weeks till 28 weeks period of gestation were screened out of which 40 GDM and 40 non GDM women were selected after performing an oral glucose tolerance test (OGTT). A TFT and anti TPO test were compared between GDM and non GDM participants. Furthermore, various parameters like sociodemographic profile, mode of delivery, pregnancy outcomes, and adverse maternal and adverse neonatal outcomes were compared.

Conclusion: The mean age of GDM women is found to be more than non GDM women. The mean TFT values are significantly lower in women with GDM as compared to non GDM women. In addition, higher values of anti TPO antibody (thyroid autoantibody) were found in the GDM group which aids in insulin resistance. Maternal complications like polyhydramnios, preterm labour, and pregnancy-induced hypertension were found to be more frequent in the GDM group compared to the non GDM group, but the results were statistically not significant. There was a higher incidence of caesarean delivery in the GDM group. Thus, we recommend the implementation of routine thyroid function profile testing in all antenatal females especially those who are at risk of developing GDM. Our study is one of the few Indian studies to evaluate the association of TFT in GDM, and we recommend similar research with a larger sample size and postnatal follow-up.

## Introduction

Gestational diabetes mellitus (GDM) is defined as glucose intolerance in a female with its onset or first recognition during pregnancy [1]. Now, the American Diabetes Association (ADA) defines GDM as diabetes that was not overt prior to pregnancy and is now diagnosed in the second or third trimesters of pregnancy [2]. Females with GDM are at higher risk of developing antenatal complications like preeclampsia during pregnancy and increased risk of type 2 diabetes as well as cardiovascular disorders later in their life. GDM has a very high prevalence rate of 11.4% in South Asian countries like India and Bangladesh [3], while the global burden of this disease remains around 14.7%. The main pathology for the development of GDM remains beta cell dysfunction along with chronic insulin resistance [4].

Maternal thyroid changes in the first and second trimesters of pregnancy have been widely related to the risk of GDM. Hypothyroidism during pregnancy is associated with early and late complications like abortions, anaemia, gestational hypertension, placental abruption and postpartum haemorrhage, impaired infant neurodevelopment, and low birth weight. They are seen more in overt hypothyroidism than in subclinical hypothyroidism. Adequate thyroid hormone levels are important for foetal neurocognitive development. Insulin resistance and GDM were found to be associated with thyroid autoimmunity and inflammation [5-9].

Various studies in the literature have compared free thyroid hormone levels in women with GDM and normal pregnant women. Significant differences in thyroid hormone levels were seen in certain studies, while others found no significant difference because the majority of them considered only a few thyroid markers, instead of the whole thyroid profile [10-15].

Our study aimed to find the association of TFT with GDM and the correlation of adverse pregnancy outcomes with TFT in GDM and non GDM women.

## Materials and methods

Our objective was to compare the thyroid function test (TFT) (serum fT3, fT4, TSH) and thyroid peroxidase antibody test (anti TPO) between GDM and non GDM pregnant women in the second trimester and to correlate the adverse pregnancy outcomes with TFT in GDM and non GDM women.

A nested case-control study was done in the Department of Obstetrics and Gynaecology, Department of Endocrinology, Department of Paediatrics, University College of Medical Sciences, and Guru Teg Bahadur (GTB) Hospital, Delhi, from November 2018 to April 2020.

Sample size

Considering the SD of 0.12 ng/dl in the case group as well as in the control group to estimate a difference of 0.13 ng/dl in the mean values of fT4, at alpha (α)=5%, and power=90%, a sample of 20 cases was required in each group. Due to the availability of time and resources, we proposed to consider 40 cases in each group. Thus, 40 cases of GDM and 40 controls were taken. Considering the 12% incidence of GDM among women visiting the antenatal clinic of GTB Hospital, 333 pregnant women needed to be screened to get 40 cases of GDM, and adding a 5% loss to follow-up, 350 pregnant women needed to be screened.

Subjects

A total of 350 pregnant women from 13 weeks to 28 weeks of gestation were recruited based on the inclusion and exclusion criteria from the antenatal clinic of GTB Hospital. A detailed history and clinical examination of all recruited women were done including age, parity, family history of diabetes, history of GDM in a previous pregnancy, history of intrauterine device/stillbirth in a previous pregnancy, history of an overweight baby in a previous pregnancy, history of smoking, and blood pressure. An oral glucose tolerance test (OGTT) was done. Results of repeat OGTT were followed, and those having OGTT less than 140 mg/dl were diagnosed as non GDM and taken as controls, and those having OGTT more than or equal to 140 mg/dl were taken in the case group. Out of the 350 recruited women, 11 were lost to follow-up. Out of the remaining 339 women, we had 44 GDM women. Four GDM cases were lost to follow-up. Hence, we had 40 GDM cases. Forty-day matched non GDM controls were randomly selected among women attending the OPD on the same day as cases. TFT and TPO antibodies were measured from the stored samples of cases and controls. The pregnancy outcomes were noted among all GDM and non GDM women. The participants were telephonically contacted and encouraged to regular initiate antenatal care visits, i.e., once every four weeks till 28 weeks of gestation, fortnightly till 32 weeks, and weekly thereafter as per WHO guidelines. The subjects were also followed up in case they visit the emergency or the labour room.

Statistical analysis

Data were compiled in Microsoft Excel (Microsoft, Washington, USA) and analysed on SPSS Statistics version 26.0 (IBM Corp. Released 2019. IBM SPSS Statistics for Windows, Version 26.0. Armonk, NY: IBM Corp.). Categorical variables were presented in number and percentage (%), and continuous variables were presented as mean ± SD and median. The normality of data was tested by the Kolmogorov-Smirnov test. If the normality was rejected, then a nonparametric test was used. Quantitative variables were compared using the independent t-test/Mann-Whitney U test between the two groups. Qualitative variables were compared using the chi-square test/Fisher’s exact test. A p-value of <0.05 was considered statistically significant.

## Results

**Figure 1:**
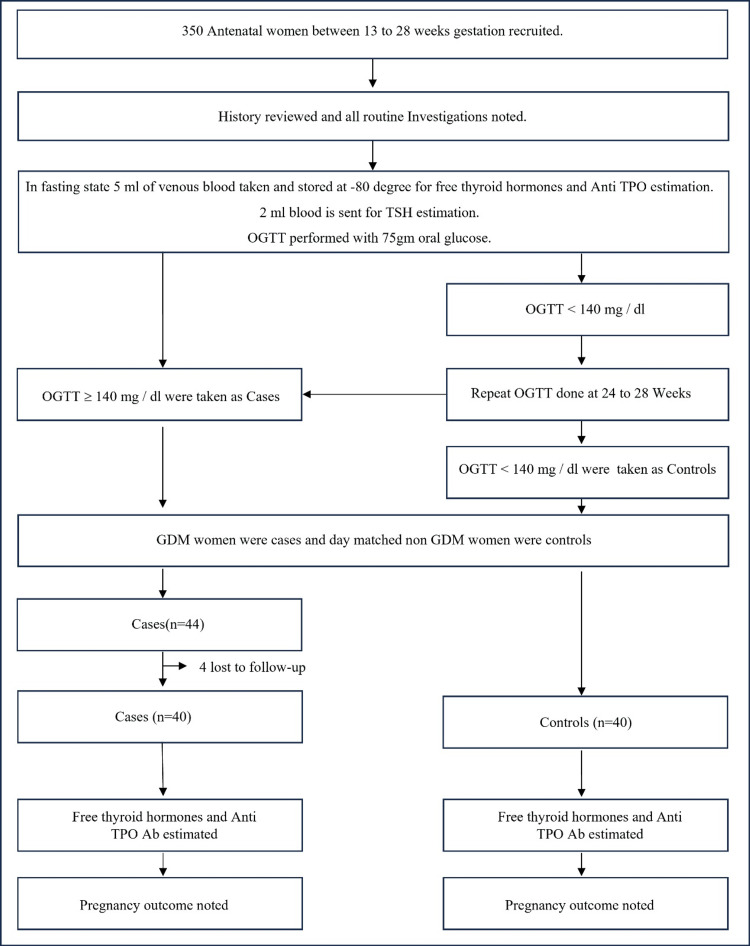
Study flow chart Anti TPO: thyroid peroxidase antibody, TSH: thyroid stimulating hormone, OGTT: oral glucose tolerance test, GDM: gestational diabetes mellitus

Figure 1 shows the study flow chat. The mean age of GDM women was 27.82 ± 3.78, and the mean age of non GDM women was 25.02 ± 2.98.17 (Table 1). There was a statistically significant difference (p=0.006)) between the age of the two groups. The incidence of GDM is seen to be increased with higher socioeconomic status (p=0.012).

**Table 1:** Frequency of distribution of sociodemographic characteristics of study subjects

		GDM (n=40)	Non GDM (n=40)	Total	p-value
Age (years)	20-25	10 (25%)	24 (60%)	34 (42.50%)	0.006
	26-30	23 (57.50%)	14 (35%)	37 (46.25%)	
	31-35	5 (12.50%)	2 (5%)	7 (8.75%)	
	36-40	2 (5%)	0 (0%)	2 (2.50%)	
Occupation	Housewife	37 (92.50%)	32 (80%)	69 (86.25%)	0.193
	Working women	3 (7.50%)	8 (20%)	11 (13.75%)	
Socioeconomic status	Lower middle	10 (25%)	23 (57.50%)	33 (41.25%)	0.012
	Upper middle	22 (55%)	14 (35%)	36 (45%)	
	Upper	8 (20%)	3 (7.50%)	11 (13.75%)	

About 97.5% of non GDM had a normal delivery, and only 2.5% had a caesarean section. About 32.5 % of GDM women had undergone a caesarean section. There was a statistically significant difference (p=0.0007) between the mode of termination of pregnancy in the two groups (Table 2).

**Table 2:** Frequency of the distribution of the parity and type of delivery of the study subjects GDM: gestational diabetes mellitus

		GDM (n=40)	Non GDM (n=40)	Total	p-value
Gravida	Gravida 1	9 (22.50%)	16 (40%)	25 (31.25%)	0.280
	Gravida 2	7 (17.50%)	7 (17.50%)	14 (17.50%)	
	Gravida 3	1 (2.50%)	0 (0%)	1 (1.25%)	
Type of delivery	Normal delivery	27 (67.50%)	39 (97.50%)	66 (82.50%)	0.0007
	Caesarean section	13 (32.50%)	1 (2.50%)	14 (17.50%)	
	Total	40 (100%)	40 (100%)	80 (100%)	

A statistically significant (p<0.001) difference was found between the OGTT values of the two groups (Table 3).

**Table 3:** Comparison of OGTT (mg/dL) between GDM and non GDM OGTT: oral glucose tolerance test, GDM: gestational diabetes mellitus

OGTT (mg/dL)	GDM (n=40)	Non GDM (n=40)	p-value
Mean ± SD	168.62 ± 21.28	97.68 ± 9.98	<0.0001

A statistical significance (p-value <0.05) was noted in the thyroid profile among the GDM and non GDM groups (Table 4).

**Table 4:** Comparison of the thyroid profile between GDM and non GDM GDM: gestational diabetes mellitus, TSH: thyroid stimulating hormone, TPO: thyroid peroxidase antibody

Thyroid profile	GDM (n=40)	Non GDM (n=40)	p-value
Free T3 (pg/dl)	2.64 ± 0.56	2.98 ± 0.51	0.006
Free T4 (ng/dl)	0.76 ± 0.42	1.26 ± 0.51	< .0001>
TSH (mIU/ml)	2.95 ± 1.1	1.33 ± 0.46	< .0001>
Anti TPO Ab (IU/ml)	22.12 ± 3.52	20.51 ± 2.87	0.027

About 87.5% of GDM women were on medical nutrition therapy (MNT), whereas the rest 12.5% of them are on both on insulin and MNT (Table 5).

**Table 5:** Frequency of the distribution of insulin + MNT/MNT of the study subjects MNT: medical nutrition therapy

Insulin + MNT/MNT	Frequency	Percentage
MNT	35	87.50%
Insulin + MNT	5	12.50%
Total	40	100.00%

There was no statistically significant difference (p=0.494) in the adverse pregnancy outcomes between the two groups (Table 6).

**Table 6:** Comparison of adverse pregnancy outcomes between GDM and non GDM GDM: gestational diabetes mellitus

Pregnancy outcomes	GDM (n=40)	Non GDM (n=40)	Total	p-value
Polyhydramnios	2 (5%)	0 (0%)	2 (2.50%)	0.494
Pregnancy-induced hypertension	4 (10%)	1 (2.50%)	5 (6.25%)	0.359
Preterm labour	2 (5%)	1 (2.50%)	3 (3.75%)	1

No statistical significance was seen in fT3, fT4, TSH, and anti TPO levels among both groups (Table 7).

**Table 7:** Comparison of the thyroid profile between subjects with and without adverse maternal outcomes GDM: gestational diabetes mellitus, TSH: thyroid stimulating hormone, TPO: thyroid peroxidase antibody

Pregnant women	Thyroid profile	With adverse maternal outcomes	Without adverse maternal outcomes	p-value
GDM	FT3 (pg/dL)	2.7 ± 0.76	2.63 ± 0.52	0.957
	FT4 (ng/dL)	0.68 ± 0.28	0.77 ± 0.44	0.762
	TSH (mIU/mL)	3.67 ± 1.55	2.8 ± 0.94	0.181
	Anti TPO (IU/mL)	23.58 ± 2.84	21.81 ± 3.61	0.23
Non GDM	FT3 (pg/dL)	3.24 ± 0.43	2.96 ± 0.51	0.335
	FT4 (ng/dL)	1.54 ± 0.78	1.24 ± 0.51	0.514
	TSH (mIU/mL)	1.83 ± 0.72	1.3 ± 0.44	0.172
	Anti TPO (IU/mL)	17.31 ± 0.47	20.68 ± 2.85	0.107

There was no statistical difference found between adverse neonatal outcomes and no adverse neonatal outcomes in the GDM group (Table 8).

**Table 8:** Comparison of the thyroid profile between subjects with and without adverse neonatal outcomes GDM: gestational diabetes mellitus, TSH: thyroid stimulating hormone, TPO: thyroid peroxidase antibody

Pregnant women	Thyroid profile	With adverse neonatal outcomes	Without adverse neonatal outcomes	p-value
GDM	FT3 (pg/dL)	2.6 ± 0.6	2.72 ± 0.59	0.624
	FT4 (ng/dL)	0.83 ± 0.51	0.7 ± 0.33	0.479
	TSH (mIU/mL)	3.32 ± 1.24	2.72 ± 1.22	0.091
	Anti TPO (IU/mL)	22.38 ± 4.06	21.37 ± 4.9	0.49
Non GDM	FT3 (pg/dL)	3 ± 0.78	2.97 ± 0.51	0.901
	FT4 (ng/dL)	1.02 ± 0.04	1.27 ± 0.52	0.732
	TSH (mIU/mL)	1.24 ± 0.11	1.27 ± 0.52	0.779
	Anti TPO (IU/mL)	16.44 ± 0.76	20.72 ± 2.78	0.037

## Discussion

Both GDM and thyroid dysfunction in pregnancy cause maternal and foetal complications. The present study compared the TFT among the GDM and non GDM women in their second trimester and compared their pregnancy outcomes and neonatal outcomes.

According to the Guidelines of the American Thyroid Association for the Diagnosis and Management of Thyroid Disease During Pregnancy and the Postpartum by Alexander et al. [16] in 2017, there is a downward shift of the TSH reference range during pregnancy, with a reduction in both the lower (decreased by about 0.1-0.2 mU/L) and the upper limit of maternal TSH (decreased by about 0.5-1.0 mU/L), relative to the typical nonpregnant TSH reference range. The largest decrease in serum TSH is observed during the ﬁrst trimester because of the elevated levels of serum hCG directly stimulating the TSH receptor and, thereby, increasing thyroid hormone production. Thereafter, serum TSH and its reference range gradually rise in the second and third trimesters but nonetheless remain lower than in nonpregnant women [16].

In our study, the average age of the GDM women was 27.82 ± 3.78, and the mean age of the non GDM women was 25.02 ± 2.98.17. There was a statistically significant difference (p=0.006) between the age of the two groups. Studies have shown that women in advanced age groups (35-39 years and >= 40 years) had a higher risk of developing gestational diabetes than the 25-29 years age group women [17-20]. We also found that the incidence of GDM was found to be increased with higher socioeconomic status. However, other studies have found an increased risk of GDM in lower-income groups [21].

Fatthy et al. [22] and Shridevi et al. [23] showed that BMI >30 was significantly associated with the risk of developing GDM. There was no statistically significant difference (p=0.633) between the BMI of the two groups in our study. This excluded BMI as a confounding factor between the two groups.

Thyroid dysfunction is one of the most common endocrine disorders affecting reproductive-age women. With time and research, gestational thyroid dysfunction is found to be an important risk factor leading to severe obstetrical complications during pregnancy or adverse pregnancy outcomes [24]. A statistically significant difference (p=0.006 and <0.001) was seen between the fT3 and fT4 levels of the two groups. Yang et al. [24] concluded that the level of free T4 in early pregnancy in GDM women is lower than non GDM women (p value<0.05). In addition, an increased level of free T4 level acts as a protective factor against the development of GDM [6]. We also found a statistically significant difference (p<0.006) between the TSH value of the two groups. Gorar et al. found that TSH levels and fT3 were significantly lower in the GDM group than in the non GDM group [25].

Thyroid autoimmunity could be an indication of autoimmune dysfunction [26]. In our study, though none of them were found to have a positive titre of antibodies, there were significantly higher values of anti TPO Ab in GDM women compared to non GDM, similar to other studies that have shown a significant association between positive thyroid antibodies and elevated TSH levels with a high risk of GDM in the second trimester of pregnancy [5,26]. Montaner et al. [27] suggested that insulin resistance is an important mechanism of thyroid antibodies for the development of GDM. The presence of thyroid antibodies results in an increase of proinflammatory cytokines, inducing insulin resistance. Nonetheless, the mechanisms involved in the association between thyroid antibodies and GDM risk remain uncertain. Yang et al. [5] found that thyroid dysfunction in pregnancy with positive thyroid antibodies might have a higher risk for GDM than thyroid dysfunction in pregnancy with negative thyroid antibodies.

We found that there was no statistically significant difference (p=0.494) between the two groups in terms of pregnancy outcomes. Five percent of GDM had polyhydramnios, and no statistically significant difference (p=0.359) was seen between the incidence of pregnancy-induced hypertension in the two groups. Preterm labour was higher (5%) among the GDM group in comparison to the non GDM group (2.5%). Complications like antepartum haemorrhage, shoulder dystocia, and stillbirth were not seen in either group. Goldman et al. [28] found that polyhydramnios and preterm labour are not significantly associated with GDM. A study conducted by Ankumah et al. [29] showed an increased incidence of preeclampsia, and shoulder dystocia is seen with increased glucose intolerance. Agarwal et al. [30] in a study measured serum fT3, fT4, and TSH in 301 pregnant women undergoing universal screening for GDM. There was no statistically significant difference in any thyroid function tests between 80 (26.6%) women with GDM and 221 (73.4%) women without GDM. Fifty-one women with anti TPO Ab positive had higher TSH than women with negative anti TPO Ab.

## Conclusions

The mean age of GDM women is found to be more than non GDM women. The mean fT3, fT4, and TSH values are significantly lower in women with GDM as compared to non GDM women. Furthermore, higher values of thyroid autoantibody were found in the GDM group which aids in insulin resistance. Maternal complications like polyhydramnios, preterm labour, and pregnancy-induced hypertension were found to be more frequent in the GDM group compared to the non GDM group, but the results were statistically not significant. There was a higher incidence of caesarean delivery in the GDM group. The participants who were diagnosed with thyroid dysfunction were started on thyroid medication during the study period. Thus, we recommend the implementation of routine thyroid function profile testing in all antenatal females especially those who are at risk of developing GDM. Our study is one of the few Indian studies to evaluate the association of thyroid function tests in GDM, and we recommend similar research with a larger sample size and postnatal follow-up.
